# Comparing Selected Life-History Traits of Black Soldier Fly (Diptera: Stratiomyidae) Larvae Produced in Industrial and Bench-Top-Sized Containers

**DOI:** 10.1093/jisesa/ieaa113

**Published:** 2020-10-22

**Authors:** Fangchun Yang, Jeffery K Tomberlin

**Affiliations:** Department of Entomology, Texas A&M University, TAMU, College Station, TX

**Keywords:** insect mass production, container size, survivorship

## Abstract

As global food demand is increasing along with human population growth, there is a greater need for alternative protein sources. Insect protein, especially the larvae of the black soldier fly, *Hermetia illucens* (L.) (Diptera: Stratiomyidae), has become a key approach for solving this issue in part due to its ability to convert organic waste into insect biomass with minimal resource (e.g., land, water) requirements. However, most information utilized to develop industrial production of this species is reliant on data generated from laboratory-scaled studies. Unfortunately, scaling these data to an industrial level potentially is not linear resulting in over, or under, estimating production. In this study, we compared selected life-history traits of larval black soldier fly produced at benchtop (e.g., 1 liter container with 614 larvae) and industrial scales (e.g., 29.5 liter container inoculated with 10,000 larvae). Larvae were provided a single feeding (2 g/larva) in each treatment. Significant differences in the mean larval weight (24.7%), survivorship (−28.2%), and biomass conversion (−2.7%) were determined between benchtop and industrial treatments. These results indicate larval number and the associated container size are important factors to consider when designing a black soldier fly factory. Furthermore, caution should be taken when applying data from laboratory studies to industrial scale production systems as the values potentially are not linear.

The global human population is rapidly expanding and is predicted to reach approximately 10 billion by 2050 (Nations 2017). In order to meet food demand, agricultural production will need to double (Tilman et al. 2011, Alexandratos and Bruinsma 2012). Protein, especially meat, is expected to have the greatest shortage due to inadequate resources needed for production. According to the World Wildlife Fund, beef alone requires more land than all other farmed animals and crops combined (WWF 2019).

A novel approach for meeting protein demands globally will be the mass production of insects. While this industry has a long cultural history (e.g., chupalinas (Orthoptera: Acridoidea) in Mexico, silkworms (Lepidoptera: Bombycidae) in China), mass production of insects for use as a feed is a more recent development (Hamamura 2001, Ramos-Elorduy 2002, Van Huis et al. 2015). In such cases, these insects can be mass produced and utilized as feed for livestock, poultry, and aquaculture (Van Huis et al. 2015). By using insects as the primary feed substrate, traditional materials (e.g., maize, soy) could be supplemented with insect protein or diverted directly to human consumption. Furthermore, some insects can be produced on materials of little to no human value, such as food waste (Nguyen et al. 2015), livestock manure (Khan et al. 2012, Koné et al. 2017), animal waste (St-Hilaire et al. 2007a), and brewery waste (Chia et al. 2018).

Insect farming can also provide environmental benefits. Feeding waste materials to insects protects air, land, and water from potential contamination (Van Huis et al. 2015). For example, the black soldier fly, *Hermetia illucens* (L.) (Diptera: Stratiomyidae), can be fed food waste that would typically be placed in landfills (Khan et al. 2012). Accordingly, digestion of these materials suppresses noxious odors (Beskin et al. 2018), greenhouse gases (Perednia et al. 2017), and pathogens (Erickson et al. 2004; Liu et al. 2008; Lalander et al. 2013, 2015). Furthermore, less land, water, and space are needed to produce insects, such as the black soldier fly, than traditional animal production (Miglietta et al. 2015). Other benefits include fast development time (e.g., black soldier fly can develop to harvestable size within 14 d) (Tomberlin et al. 2002) versus beef (e.g., 12–18 mo of feeding to reach the needed weight to slaughter) (WWF 2019). It is also worth noting that the full insect is edible unlike beef (48.5%) (Miglietta et al. 2015). Because of the ability of the black soldier fly to consume a variety of organic wastes, while offering benefits to the environment, it is now viewed as the ‘crown jewel’ of the insects as feed industry and is mass produced globally (Tomberlin and van Huis 2020), while also being approved for use as poultry feed in the United States and Canada as well as feed for select aquaculture species in the same locations as well as the European Union (AAFCO 2019, Einstein-Curtis 2019, IPIFF 2020).

Current knowledge about this species has been largely developed based on lab-scale (i.e., benchtop) studies (Diener et al. 2009, Paz et al. 2015, Beskin et al. 2018), including those major findings that have served as milestones in the black soldier fly farming industry (Sheppard et al. 2002; Tomberlin et al. 2002, 2009). For example, methods developed for mass production of this insect in colony were developed using 300 ml containers (Sheppard et al. 2002). While these methods serve as the cornerstone of all companies globally mass producing the black soldier fly, determining how to apply them at a larger scale is a challenge. The same can be said for other studies examining the conversion of waste into insect biomass (Lalander et al. 2019). In most instances, they were accomplished using small containers (e.g., 14 × 7.5 × 7 cm; Diener et al. 2009), which are not necessarily the size used in mass production. For example, industrial settings often use containers at least 10× larger (F. Yang, unpublished data).

Ultimately, scale matters when applying data from a small study to a much larger setting (McGill 2010). For example, with the black soldier fly, the temperature in a 665 × 435 × 160 mm plastic bin with actively feeding larvae can be well above room temperature reaching 42°C (F. Yang, unpublished data), which is not common with benchtop scale where containers are typically remain at ambient temperature (Meneguz et al. 2018b). Consequently, many ‘in house’ studies by industry are necessary as a means to optimize black soldier fly production. Unfortunately, such data in many cases are private and have not been validated through open research channels. The objective of this study was to demonstrate the differences between benchtop and industrial scale with regards to black soldier fly production and waste conversion to insect biomass. By knowing whether the black soldier fly larvae exhibit different life-history traits at different scales, industry can better design production systems that optimize production and waste conversion.

## Materials and Methods

### Industrial Site Process

Experiments were conducted in a black soldier fly production facility designed and managed by JM Green Environmental Protection Ltd in Baotou, Inner Mongolia Province, China. The facility is part of a waste management project in Baotou, which is designed to recycle 100 metric tons restaurant kitchen waste per day.

Organic waste collected from local restaurants was used in the experiments. Upon delivery at the factory, the organic wastes, collected within 24 h, were first processed through an automatic sorter to remove nonfood waste items, such plastic bottles and aluminum cans. Remaining waste is then ground into a slurry form with a particle size under 5 mm. The slurry was then cooked for 4 h at 80°C before being processed through a centrifuge which produced three materials: lipids, liquids, and solids. The lipid phase was collected for sale as an ingredient for bio-diesel production, and the liquids are processed through a biogas reactor. There was usually 20 metric tons of remaining solids with 70–80% water content from a batch of 100 metric tons of raw waste, and these solid wastes were used for black soldier fly larval feedstock.

Prior to digestion with black soldier fly larvae, the solid sludge was placed in intermediate bulk containers (IBC tanks, 1,000 liters) and inoculated with 30 liters of *Lactobacillus* culture and allowed to ferment for at least 24 h. After fermentation, which was indicated by the pH dropping from 6 to below 4, sludge, the material, now known as feedstock, was transferred into a feed mixer where ad lib amounts of wheat bran were added to reduce moisture to 70%. Once moisture content is adjusted, feedstock is pumped into growing pans, which are described below to feed the larvae.

### Black Soldier Fly Population

One kilogram of black soldier fly eggs was received from a facility in Guangdong Province, China. Eggs were partitioned into 50 g allotments and placed on metal stand (20 cm in diameter) covered with a screen mesh (1.5 mm) and positioned 5 cm above a neonate larval substrate in a 665 × 435 × 160 mm blue plastic bin. The neonate larval substrate was made with 50% fermented food waste prepared as aforementioned, and 50% wheat bran by volume, with water added to adjust moisture to 70%. Doing so allowed neonates to drop down to the tray and have immediate access to the feedstock. Trays were stored in a rearing room at 28°C, 60–90% relative humidity, and 8:16 (L:D) h. Neonates were allowed to feed for 4–5 d. Larvae were then separated with a 2 mm sifter and used to inoculate industrial pans containing feedstock (described subsequently).

### Experiment Design

**Fig. 1.**
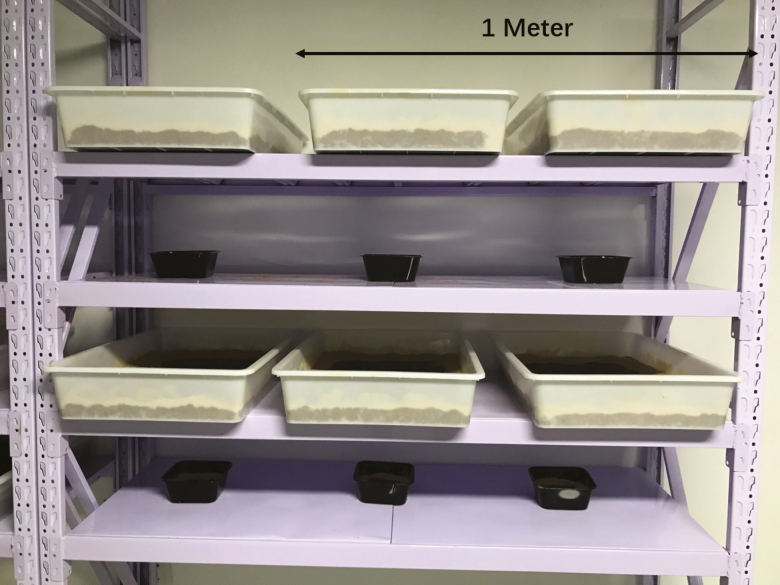
Setup for industrial and small-scale black soldier fly larval production. Pans containing feedstock and black soldier fly larvae were randomly placed on shelves in a rearing room 27–30°C, 60–90% RH, 8:16 (L:D) h.

Two trials of the following experiment were conducted. For each trial, six replicates were used for each treatment. For the industrial treatment, which is based in part on production in the Baotou facility, each replicate consisted of a 29.5 liter white plastic tray (610 × 420 × 115 mm). For small-scale (benchtop), a 1 liter plastic tray (155 × 155 × 75 mm) was used as a replicate. Large-scale containers were filled with 5 kg feedstuff at the beginning (i.e., single feeding) of the experiment and inoculated with 10,000 larvae (two larvae per gram of feedstuff). Each small-scale container was filled with 307 g feedstuff and inoculated with 614 larvae (2 larvae per gram of feed stuff). Accounting for amount of food provided, allowed for feed amount per larva to be excluded as a factor. The feed amounts were determined to ensure the same amount of feed was placed per cm^2^ so that the depths of the substrate were equal between these two containers. Both treatments were placed on the same rack for processing (Fig. 1).

All experiments were conducted at 27–30°C, 60–90% RH, 8:16 (L:D) h. Observations were made every 24 h. On the 7th day of the experiment (harvesting point in factory) for industrial and small-scale treatments, larvae, and frass from each tray/container were separated using a 5 mm mesh sieve.

Three parameters commonly monitored in production facilities, and thus recorded in this study, are mean larval weight, larval survivorship, and the larval conversion rate. Five subsamples of 10 larvae were randomly collected from each replicate and weight recorded in order to determine mean larval weight. Larval survivorship was calculated by using the total weight of the harvested larvae divided by the mean larval weight. Larval mass conversion rate was calculated by using total larval weight divided by feed amount given.

### Data Collection and Analysis

Following confirmation that data for a given variable measured met parametric requirements, an analysis of variance (ANOVA) was performed (RStudio, version 1.1.383) followed by a Tukey’s HSD (honest significant difference) to determine mean separation (*P* ≤ 0.05).

## Results

**Fig. 2.**
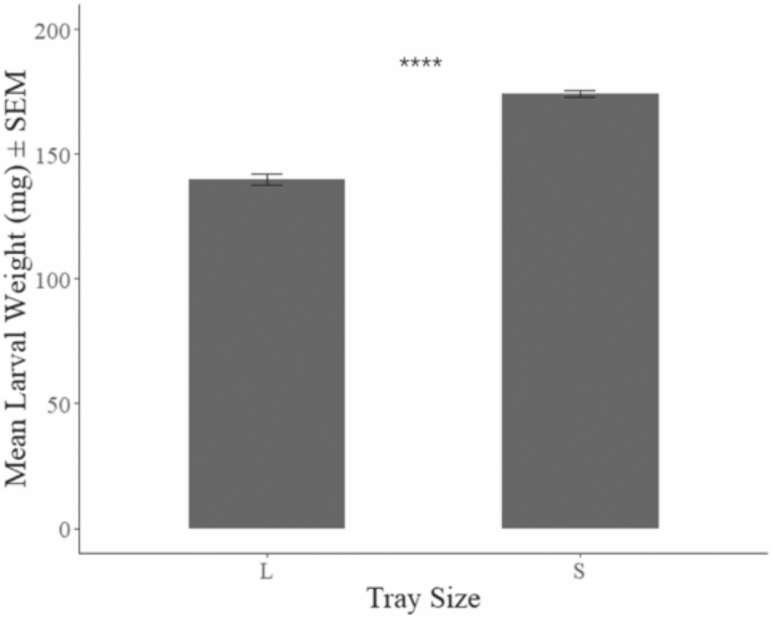
Mean larval mass weight ± SEM after 7 d of rearing in different size containers placed in 27–30°C, 60–90% RH environment, with 8:16 (L:D) h photoperiod. ****ANOVA test result indicates mean values from the two treatments are significantly different with *P* < 0.05.

**Fig. 3.**
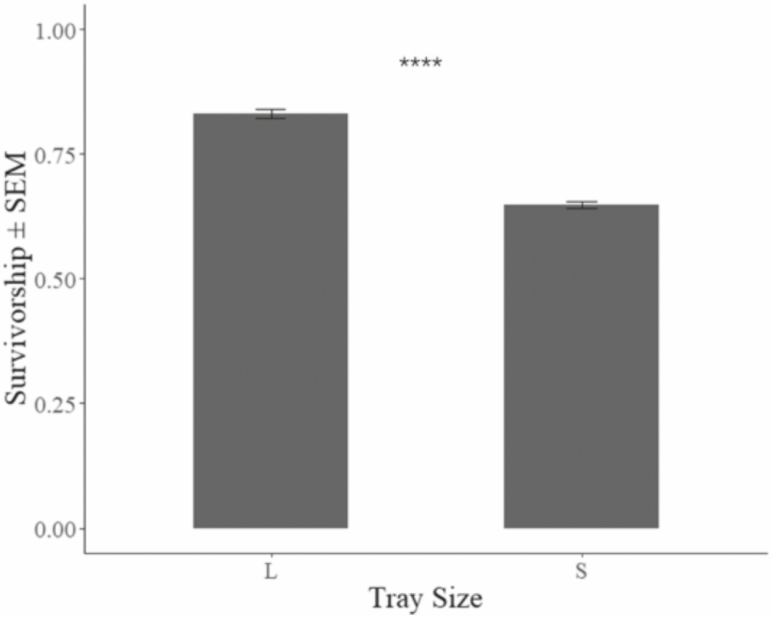
Larval survivorship ± SEM after 7 d of rearing in different size containers placed in 27–30°C, 60–90% RH environment, with 8:16 (L:D) h photoperiod. ****ANOVA test result indicates mean values from the two treatments are significantly different with *P* < 0.05.

**Fig. 4.**
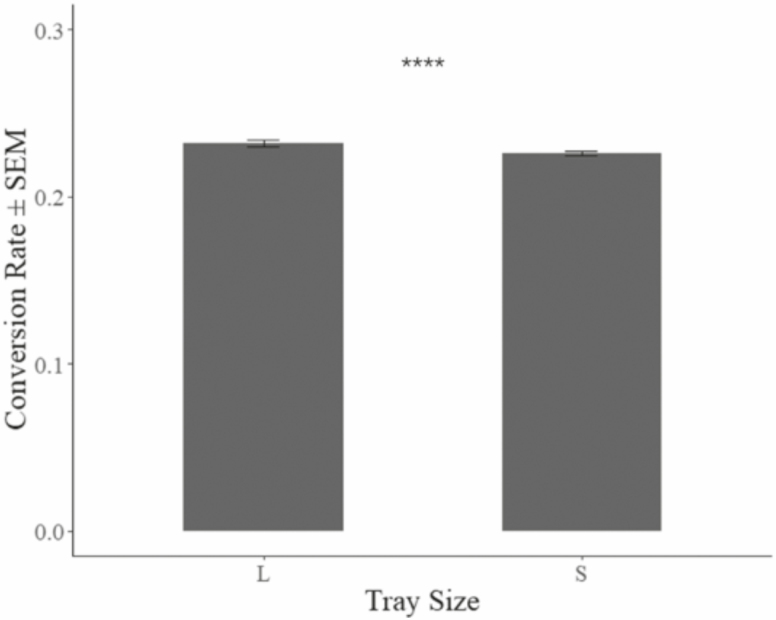
Larval mass conversion rate ± SEM after 7 d of rearing from different containers placed in 27–30°C, 60–90% RH environment, with 8:16 (L:D) h photoperiod. The conversion rate was calculated with the total weight of larvae harvested at the end divided by the weight of the feed started in the respective containers. ****ANOVA test result indicates mean values from the two treatments are significantly different with *P* < 0.05.

Final mean larval weight (industrial = 139.87 mg ± 7.65 mg, benchtop = 174.41 mg ± 1.35 mg) of black soldier fly larvae (Fig. 2) resulting from the benchtop treatment was 24.7% greater than in the industrial treatment (F_1,22_ = 177.7, *P* < 0.01). However, survivorship (industrial = 83.13 ± 3.25%, benchtop = 64.78 ± 2.31%) in the industrial treatment was 28.2% greater (Fig. 3) than in the benchtop treatment (F_1,22_ = 253.1, *P* < 0.01). And, larval mass conversion rate (industrial = 23.21 ± 0.69%, benchtop = 22.58 ± 0.56%) was 2.7% greater (Fig. 4) in the industrial treatment (F_1,22_ = 5.99, *P* < 0.05). An assessment of the economic impact (Table 1) of these values has been determined based on industry experience (F. Yang, unpublished data) in China.

**Table 1.** Projection of amount of waste and eggs needed in order to produce 100 metric tons of black soldier fly larvae using industrial large-scale data versus benchtop small-scale data

	L^*a*^	S^*b*^	% Difference
Amount of Waste (metric ton)	431	442	2.6%
Amount of Eggs^*c*^ (kg)	20.1	25.7	27.9%

^*a*^L = 83.1% survivorship, larval size = 0.15 g each, conversion rate = 23.2%.

^*b*^S = 64.8% survivorship, larval size = 0.15 g each, conversion rate = 22.6%.

^*c*^Each gram of black soldier fly egg contains 40,000 individuals (Cammack, unpublished data).

## Discussion

Data produced through this study have tremendous implications for the value of benchtop data being applied by industry. At face value, these data would indicate the application of the benchtop data to an industrial production facility could grossly under-estimate production levels and conversion rates. If we were to translate benchtop data from the current study to industrial scale (i.e., system used in current study is applied in the industrial facility where the study took place), predictions would be inaccurate as far as production and thus financially. For example, using the data from the benchtop treatment to design a factory aiming to produce 100 metric tons of larvae with an average larval size of 0.15 g, they would require 5.6 kg (28%) more eggs, which equals to $5,600 at current market price (F. Yang, unpublished data), in order to account for high mortality (Table 1).

In fact, larval conversion rates across the two treatments further emphasize this point. Larval mass conversion rate at the industrial scale large trays was 0.6% greater, which was statistically significant, than that at the benchtop scale. While unassuming, such a small difference translates into massive economic impact. For example, the factory used as the study site digests 20 tons of feedstuff daily with black soldier fly larvae. If the 0.6% were translated to this scale, it would equate to the additional production of 120 kg of fresh larvae, which equals to 40 kg of dried product, every day. If extrapolated to 1 yr, 0.6% increased production would yield 14,600 kg of dried product valuing over USD $250,000 in the U.S. retail market as of 2019. Furthermore, the 0.6% greater conversion rate also means 30 m^3^ less frass a year (F. Yang, unpublished data), and in some cases this is preferred as it is seen as byproduct of less value than the resulting larvae.

Producing larger larvae in a facility could reflect low survivorship as observed in this study. Larvae reared at the benchtop scale suffered greater mortality (Fig. 3) than those in the industrial treatment. Consequently, those that survive experience less competition for resources. This result does not come as a surprise as it has been observed for other species. For example, adult size of *Lucilia cuprina* (Wiedemann) (Diptera: Calliphoridae), which is a carrion colonizer, oscillated over generations when restricted to a set amount of feed (Nicholson 1950). The initial generation over-populated the carrion resulting in almost 100% mortality and 50% smaller flies being produced. Given the adults of the second generation were small, they under-populated the fresh carrion source (i.e., same size and quality as with the first generation) resulting in high survivorship and large adults. The same was observed in the current study. The largest larvae produced were from replicates that suffered the greatest mortality (i.e., reduced competition), while the smaller larvae were from the industrial replicates where mortality was lower (i.e., greater competition for food) (Figs. 2 and 3).

Defining the optimal larval density and associated feed rate within an environment (i.e., pan dimensions and food allocation) are critical for maximizing larval survival and subsequent production. As demonstrated in this study, smaller group of larvae in the smaller container resulted in less ideal survivorship, and this is not restricted to black solider fly, as described with *L. cuprina*. The suboptimal density impact insect survivorship in both ways, when higher larval density resulted in lower survivorship, opposite results were also determined with the butterfly *Euselasia chrysippe* (Bates) (Lepidoptera: Riodinidae), where two times bigger group size resulted in 21.6 times greater survivorship (Allen 2010).

In some instances, greater larval number, to a degree, for some species can be beneficial. For example, greater larval density can suppress pathogen proliferation, such as with *Drosophila melanogaster* Meigen (Diptera: Drosophilidae), where fungi are known to grow on larval resources if the larval density is not above a given threshold (Wertheim et al. 2002). Optimal densities can also allow for thermoregulation which is critical for development. Larval *L. curprina* aggregation and feeding can result in a microclimate 15°C above ambient conditions, which allows them to develop two times faster than those at a lower density (Kotzé et al. 2016).

Such modifications of the environment (i.e., larval number, pan size, feed rate) could be critical for black soldier fly production. Under more natural conditions, black soldier fly larvae occur at high larval numbers in dung piles (Sheppard 1983, Fatchurochim et al. 1989) or carrion (Tomberlin et al. 2005), which are normally associated with unpredictable pathogen levels (Diclaro and Kaufman 2009). The ability of black soldier fly larvae to modify pH (Ma et al. 2018, Meneguz et al. 2018a) can result in suppressed pathogens (Wang et al. 2015) and improve the palatability of the food (Deshpande et al. 2015). Furthermore, black soldier fly larvae produce antimicrobial peptides that can inhibit a broad spectrum of bacteria (Park et al. 2014, 2015; Vogel et al. 2018), and studies have shown that black soldier fly larvae can reduce *Escherichia coli* (Erickson et al. 2004, Liu et al. 2008), *Salmonella* spp. (Erickson et al. 2004; Lalander et al. 2013, 2015), and phage (Lalander et al. 2015) in the waste. However, such processes are potentially partially regulated by larval number thus explaining the greater mortality in the low number, benchtop, treatment rather than the industrialized scale examined in the current study.

While not examined in the current study, it should be noted that the larger larvae produced at the benchtop scale may not equate quality larvae. Nutrient content of larvae can vary depending on size. Protein content is key factor regulating the use of black soldier fly larvae as animal feed (Bondari and Sheppard 1981, 1987; St-Hilaire et al. 2007b; Sealey et al. 2011; Widjastuti et al. 2014). However, black soldier fly larvae shift from protein to fat accumulation as they age (Liu et al. 2017). Although they only investigated nutrient content shift in relationship to larval age, other researchers have found lipid content increased exponentially with body size for other species. For example, protein content increased linearly with the growth of the yellow fever mosquito, *Aedes aegypti*, Linnaeus *in* Hasselquist, (Diptera: Culicidae); however, lipid accumulation was more exponential suggesting larger larvae would have more fat than smaller conspecifics (Timmermann and Briegel 1999). If the same occurs for black soldier fly larvae, then larger larvae potentially are not ideal due to their high fat content.

This study demonstrated select life-history traits of black soldier fly larvae are impacted based on pan size (benchtop vs industrial) with the same larval density. Standardizing the rearing containers for black soldier fly larvae production is critical for optimal production. Furthermore, while valuable, laboratory studies only scratch the surface in terms of elucidating the factors regulating larval growth and production. Future studies should consider such scale issues when evaluating industrial value of results generated and making recommendations.
